# Non-Coding SNPs Regulate Bovine Muscle Satellite Cell Proliferation and Differentiation by Modulating *PENK* Expression

**DOI:** 10.3390/ijms27167467

**Published:** 2026-08-20

**Authors:** Tianyi Wu, Feng Liu, Qunhao Niu, Zhida Zhao, Lupei Zhang, Huijiang Gao, Junya Li, Xue Gao, Lingyang Xu

**Affiliations:** 1 State Key Laboratory of Animal Biotech Breeding, Institute of Animal Sciences, Chinese Academy of Agricultural Sciences (CAAS), Beijing 100193, China; tianyi_wu163@163.com (T.W.);; 2 Northern Agriculture and Livestock Husbandry Technology Innovation Center, Chinese Academy of Agricultural Sciences (CAAS), Hohhot 010111, China

**Keywords:** *PENK*, noncoding variant, bovine muscle satellite cell, CRISPR, muscle growth

## Abstract

The functions of noncoding variants associated with complex traits in livestock remain poorly understood. In this study, we investigated two candidate noncoding variants within the *XKR4-CHCHD7* locus identified from our previous analysis. Dual-luciferase reporter assays demonstrated allele-specific regulatory activity of these two regions in bovine muscle satellite cells (BMSCs), 293T cells, and C2C12 cells. Endogenous deletion of the candidate regions using a clustered regularly interspaced short palindromic repeats (CRISPR)-based high-fidelity Cas12Max (hfCas12Max) system revealed that the region containing chr14:22840845 (SNP-0845) exerted broader effects on BMSC function including reduced proliferation and migration, altered cell-cycle progression, and enhanced myogenic differentiation. Expression screening of candidate effector genes further identified *PENK* and *TMEM68* as downstream candidate genes for SNP-0845. Rescue experiments further showed that *PENK* exerted stronger recovery effects than *TMEM68* on the proliferation and migration defects caused by deletion of this region, supporting *PENK* as a major candidate effector downstream of the SNP-0845. Functional assays showed that *PENK* knockdown impaired BMSC proliferation and migration while promoting myogenic differentiation, whereas *PENK* overexpression partially reversed these effects. In vivo *Penk* knockdown reduced quadriceps femoris weight and altered muscle fiber composition in mice. Collectively, our findings suggest that a noncoding regulatory region modulates BMSC fate and muscle growth-related processes through *PENK*.

## 1. Introduction

Muscle growth and development are important traits in beef cattle breeding, directly impacting meat production performance and farming profitability [1]. With the development of high-density genotyping and sequencing technologies, numerous quantitative trait loci (QTLs) associated with body size, carcass traits, and meat yield in cattle have been identified [2,3,4]. However, many trait-associated variants are located in noncoding regions, making it difficult to interpret their biological functions and limiting their direct application in molecular breeding [5].

Noncoding variants may influence complex traits by altering cis-regulatory elements, transcription factor binding, chromatin accessibility, or long-range enhancer–promoter interactions [6]. Therefore, functional validation of candidate noncoding variants is essential for moving from statistical association to biological mechanism [7]. Reporter assays can provide initial evidence of allele-specific regulatory activity, whereas genome editing enables evaluation of candidate regulatory regions in their endogenous genomic context [8]. In livestock, functional validation of noncoding variants remains challenging because relevant primary cells often have limited proliferative capacity [9] and relatively low genome-editing efficiency.

Muscle satellite cells are the major stem cell population responsible for postnatal skeletal muscle growth, repair, and regeneration [10]. The balance between satellite cell proliferation, migration, and myogenic differentiation is critical for muscle development and muscle mass maintenance [11]. Therefore, bovine muscle satellite cells (BMSCs) provide a biologically relevant model for investigating how trait-associated regulatory variants affect muscle growth-related cellular processes [12].

Our previous genome-wide association study (GWAS)-based analysis identified five candidate noncoding single-nucleotide polymorphisms (SNPs) within the *XKR4-CHCHD7* locus on *Bos taurus* chromosome 14 (BTA14) [4]. This locus has also been repeatedly associated with carcass weight and meat-cut traits in cattle [13,14], and contains several genes with possible roles in growth and cellular regulation, including *PENK* and *TMEM68*.

*PENK* encodes the proenkephalin precursor, which is processed into multiple bioactive enkephalin peptides. Although *PENK* has been studied predominantly in the nervous and endocrine systems, several studies have suggested a potential role in skeletal muscle biology. Early developmental studies detected proenkephalin expression in mesodermal lineages and neonatal skeletal muscle [15,16], and subsequent work demonstrated enkephalin immunoreactivity and release from skeletal muscle, indicating that the *PENK* system is also active in peripheral muscle tissues [17]. More directly, Nihashi et al. reported that *PENK* expression decreased during myogenic differentiation in chicken myoblasts, with similar differentiation-dependent decreases observed in primary mouse and human myoblasts. In the same study, exogenous methionine-enkephalin and leucine-enkephalin suppressed chicken myoblast proliferation but did not significantly affect myotube differentiation [18]. In livestock, *PENK* has also been implicated in muscle-related traits through genetic association and transcriptomic analyses [19,20]. However, these previous studies have largely focused on developmental expression, tissue-level associations, or the effects of exogenously administered enkephalin peptides. Whether endogenous *PENK* directly regulates the proliferation–differentiation balance of bovine muscle satellite cells remains unclear, and no functional link has been established between *PENK* and a trait-associated noncoding regulatory region in cattle.

In this study, we comprehensively investigated the functional roles of SNPs within the BTA14 *XKR4-CHCHD7* locus. We first evaluated the allele-specific regulatory activity of chr14:22840845 and chr14:22999212 using dual-luciferase reporter assays and performed clustered regularly interspaced short palindromic repeats (CRISPR)-based high-fidelity Cas12Max (hfCas12Max)-mediated deletion of the corresponding regions containing the candidate SNP in primary BMSCs. Then, using expression screening, gene perturbation, rescue assays, and in vivo *Penk* knockdown, we identified *PENK* as a major candidate effector downstream of the regulatory region containing chr14:22840845. Our study provides functional evidence linking a noncoding regulatory region to BMSC fate regulation and muscle growth, offering new insight into the biological interpretation of noncoding variants for complex traits in farm animals.

## 2. Results

### 2.1. Functional Screening Prioritizes SNP-0845 as a Regulatory Region Controlling BMSC Fate

**Figure 1:**
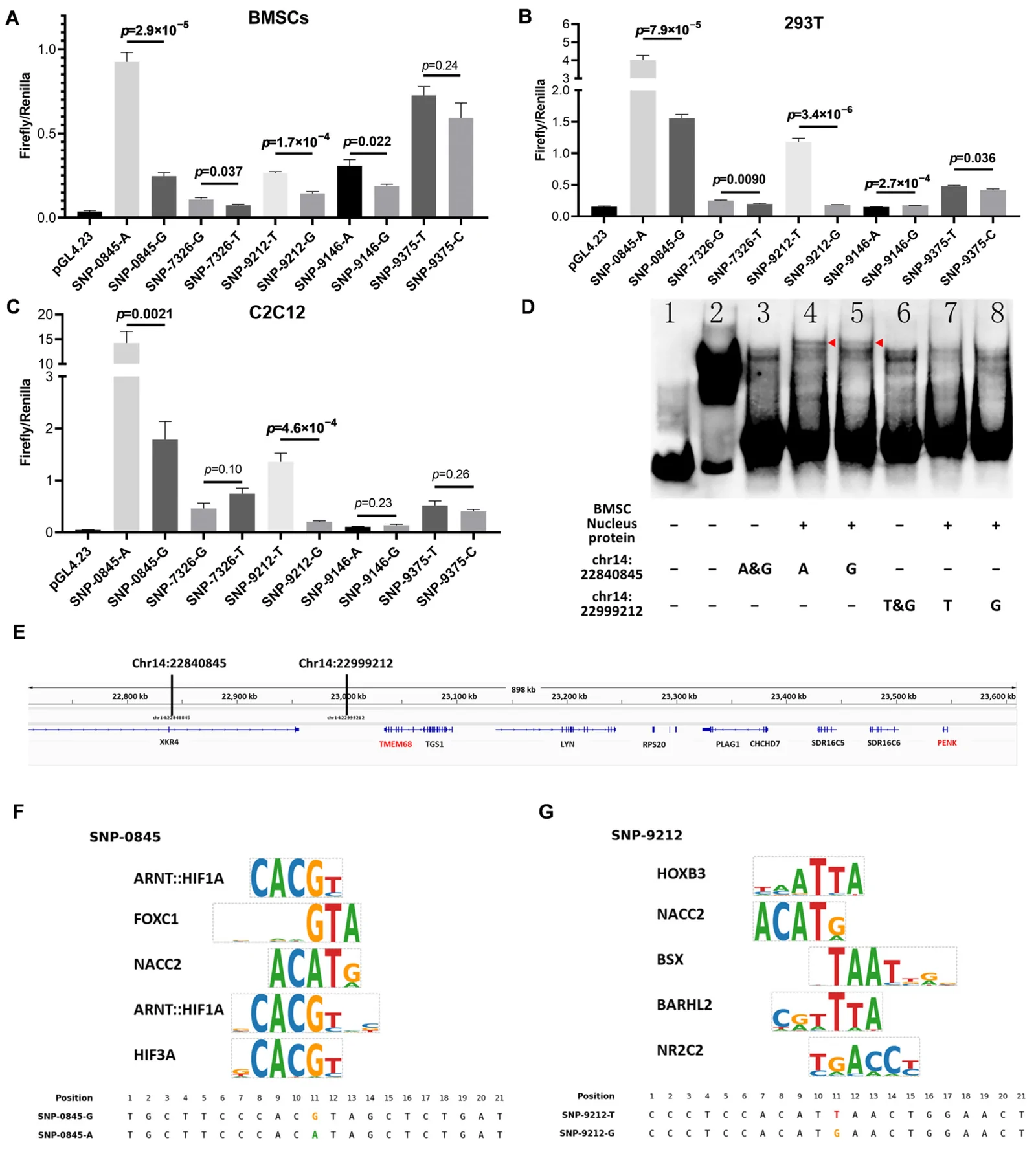
Allele-specific regulatory activity and phenotypic relevance of candidate single-nucleotide polymorphisms (SNPs) within the *Bos taurus* chromosome 14 (BTA14) locus. (**A**–**C**). Dual-luciferase reporter assay results for allelic variants of SNPs in bovine muscle satellite cells (BMSCs) (**A**), 293T cells (**B**), and C2C12 cells (**C**) (*n* = 4). (**D**) Preliminary electrophoretic mobility shift assay (EMSA) results for probes containing SNP-0845 or SNP-9212. Nuclear extracts from BMSCs were used for the binding assay (*n* = 1). Lane 1 contains the positive probe only; lane 2 contains the positive probe with positive protein; the remaining lanes are as indicated in the notes below the image, where the letters “A”, “G”, “T”, etc., denote probes corresponding to the respective genotypes at each locus. The red arrows indicate the unique bands of the SNP-0845 probes. (**E**) Schematic diagram of the genomic locations of SNP-0845 and SNP-9212, showing the positions of major genes within the chr14:22,800 kb–23,550 kb region. (**F**) Representative transcription factor-binding motifs predicted to be affected by the A/G allelic substitution at SNP-0845. (**G**) Representative motifs predicted to be affected by the T/G allelic substitution at SNP-9212. *p*-values are highlighted in bold font to indicate significant differences and these indicators are used consistently throughout the manuscript. Candidate SNPs were designated as follows: SNP-0845 (chr14:22840845), SNP-7326 (chr14:22867326), SNP-9212 (chr14:22999212), SNP-9146 (chr14:23029146), and SNP-9375 (chr14:23329375).

To functionally screen the five candidate noncoding SNPs identified in our previous analysis (Supplementary Table S1) [4], approximately 500 bp genomic fragments centered on each allele were cloned into luciferase reporter vectors. (Supplementary Figure S1). The effects of allelic variants on relative firefly luciferase activity (normalized to *Renilla* luciferase) were examined in three cell types: BMSCs (Figure 1A), 293T cells (Figure 1B), and C2C12 cells (Figure 1C). Two SNPs, referred to as SNP-0845 and SNP-9212 (at chr14:22840845 and chr14:22999212) exhibited consistent and highly significant allelic differences in luciferase activity across all three cell types. Preliminary electrophoretic mobility shift assay (EMSA) further revealed distinct DNA–protein binding patterns between the two candidate regions. Probes containing SNP-0845 produced an additional shifted band (red arrows) that was not observed for SNP-9212, whereas other shifted bands were similar between the two loci (Figure 1D). These observations suggested that the SNP-0845-containing sequence may harbor a more distinctive nuclear protein-binding site, providing additional support for prioritizing SNP-0845 in subsequent functional analyses. A schematic of the *XKR4-CHCHD7* locus further illustrated the genomic positions of SNP-0845 and SNP-9212 relative to nearby genes (Figure 1E).

To further explore whether SNP-0845 and SNP-9212 may alter potential transcription factor (TF)-binding motifs, we performed an in silico analysis using the JASPAR 2026 database. Multiple TF-binding motifs overlapping the variant positions were predicted at a relative profile score threshold of 80%. For each SNP, the five motifs with the highest relative scores were selected for further allele-specific comparison, and the corresponding relative scores for the alternative allele were calculated using the same JASPAR matrices (Supplementary Table S2). These representative motifs showed marked allele-dependent differences in predicted motif scores (Figure 1F,G). At SNP-0845, the largest differences were observed for ARNT::HIF1A, FOXC1, NACC2, and HIF3A motifs, whereas at SNP-9212, pronounced differences were observed for HOXB3, NACC2, BSX, BARHL2, and NR2C2 motifs. These results suggest that both variants may alter local TF-binding motif compatibility and provide computational support for their potential regulatory effects.

### 2.2. Endogenous Perturbation Prioritizes the SNP-0845 Region as a Functional Regulator of BMSC Fate

**Figure 2:**
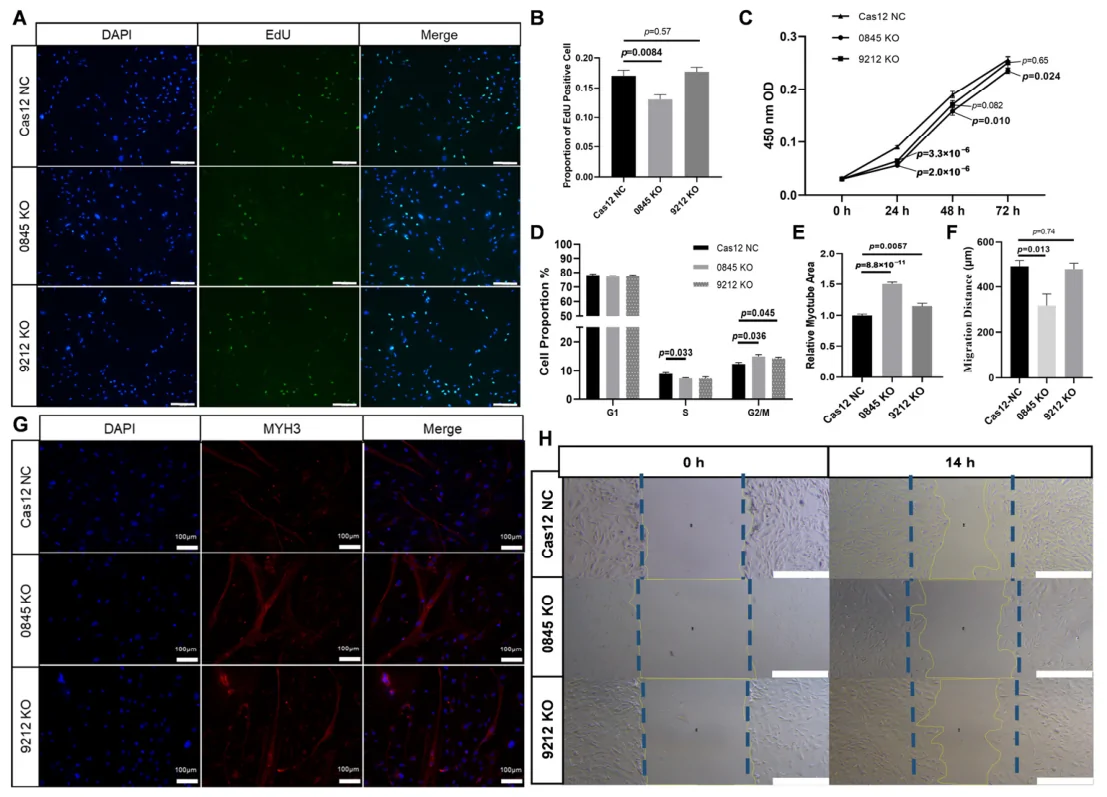
Functional consequences of clustered regularly interspaced short palindromic repeats (CRISPR)/hfCas12MAX-mediated editing of the region containing SNP-0845 or SNP-9212 in BMSCs. (**A**) Representative 5-ethynyl-2′-deoxyuridine (EdU) staining images 48 h after editing of the region containing SNP-0845 or SNP-9212. (**B**) Quantification of EdU-positive cells from the experiments shown in (**A**) (*n* = 6). (**C**) Cell Counting Kit-8 (CCK-8) assay measuring cell viability at 0, 24, 48, and 72 h after CRISPR-mediated editing (*n* = 6). (**D**) Cell-cycle analysis by flow cytometry using PI staining 48 h after editing (*n* = 3). (**E**) Quantification of myogenic differentiation from the experiments shown in (**G**). Myogenic differentiation capacity was assessed as myotube area normalized to nuclei number (*n* = 6). (**F**) Quantification of wound-healing assay results from the experiments shown in (**H**) (*n* = 6). (**G**) MYH3 immunofluorescence staining of myotubes after 4 days of differentiation following editing of the region containing SNP-0845 or SNP-9212. (**H**) Representative images of wound-healing assays following editing of the region containing SNP-0845 or SNP-9212. Blue dashed lines outline the wound edge at 0 h. Scale bars: 200 μm (**A**), 100 μm (**G**), and 500 μm (**H**). 0845 KO, BMSCs with deletion of the region containing SNP-0845 using 0845 crRNA1 and 0845 crRNA2; 9212 KO, BMSCs with editing of the region containing SNP-9212 using 9212 crRNA1; Cas12 NC, BMSCs transfected with equivalent amounts of hfCas12MAX plasmid.

To investigate whether the genomic regions containing SNP-0845 and SNP-9212 contribute to BMSC function, we established CRISPR/hfCas12MAX-mediated deletion strategies for both regions (Supplementary Figure S2A). Efficient deletion of the regions containing SNP-0845 and SNP-9212 was confirmed by sequencing (Supplementary Figure S2B,C). We first assessed the impact of these regional deletions on BMSC proliferation. 5-ethynyl-2′-deoxyuridine (EdU) incorporation assays were performed 48 h after flow cytometry sorting. Deletion of the region containing SNP-0845 significantly reduced EdU incorporation compared with the control group (*p* < 0.01), whereas deletion of the region containing SNP-9212 had no significant effect (*p* > 0.05) (Figure 2A,B). Cell Counting Kit-8 (CCK-8) assays further confirmed a sustained reduction in cell viability after deletion of the region containing SNP-0845, whereas the effect of deletion of the region containing SNP-9212 was transient and weaker (Figure 2C). Similar results were observed in an independent BMSC clone carrying a 73 bp deletion within the SNP-0845-containing region (Supplementary Figure S3). Cell-cycle analysis using propidium iodide (PI) staining and flow cytometry showed that deletion of the region containing SNP-0845 increased the proportion of cells in G2/M phase and reduced the S-phase population, suggesting impaired cell-cycle progression. Deletion of the region containing SNP-9212 also increased the G2/M-phase population, but the overall effect was weaker (Figure 2D). We next examined myogenic differentiation by immunofluorescence staining. Deletion of the region containing SNP-0845 significantly enhanced MYH3-positive myotube formation (*p* < 0.001), while deletion of the region containing SNP-9212 also promoted differentiation to a lesser extent (Figure 2E,G). We further examined BMSC migration using wound-healing assays. Deletion of the region containing SNP-0845 significantly reduced wound closure (*p* < 0.05), whereas deletion of the region containing SNP-9212 had no significant effect (*p* > 0.05) (Figure 2F,H). Taken together, the more consistent and stronger phenotypic effects observed upon deletion of the region containing SNP-0845-including sustained reduction in BMSC proliferation and migration, greater cell-cycle perturbation, and more pronounced enhancement of myogenic differentiation—compared with the weaker and more transient effects observed for the region containing SNP-9212, led us to prioritize SNP-0845 for downstream mechanistic dissection.

### 2.3. Expression Screening Identifies PENK and TMEM68 as Candidate Effector Genes Downstream of the SNP-0845 Region

**Figure 3:**
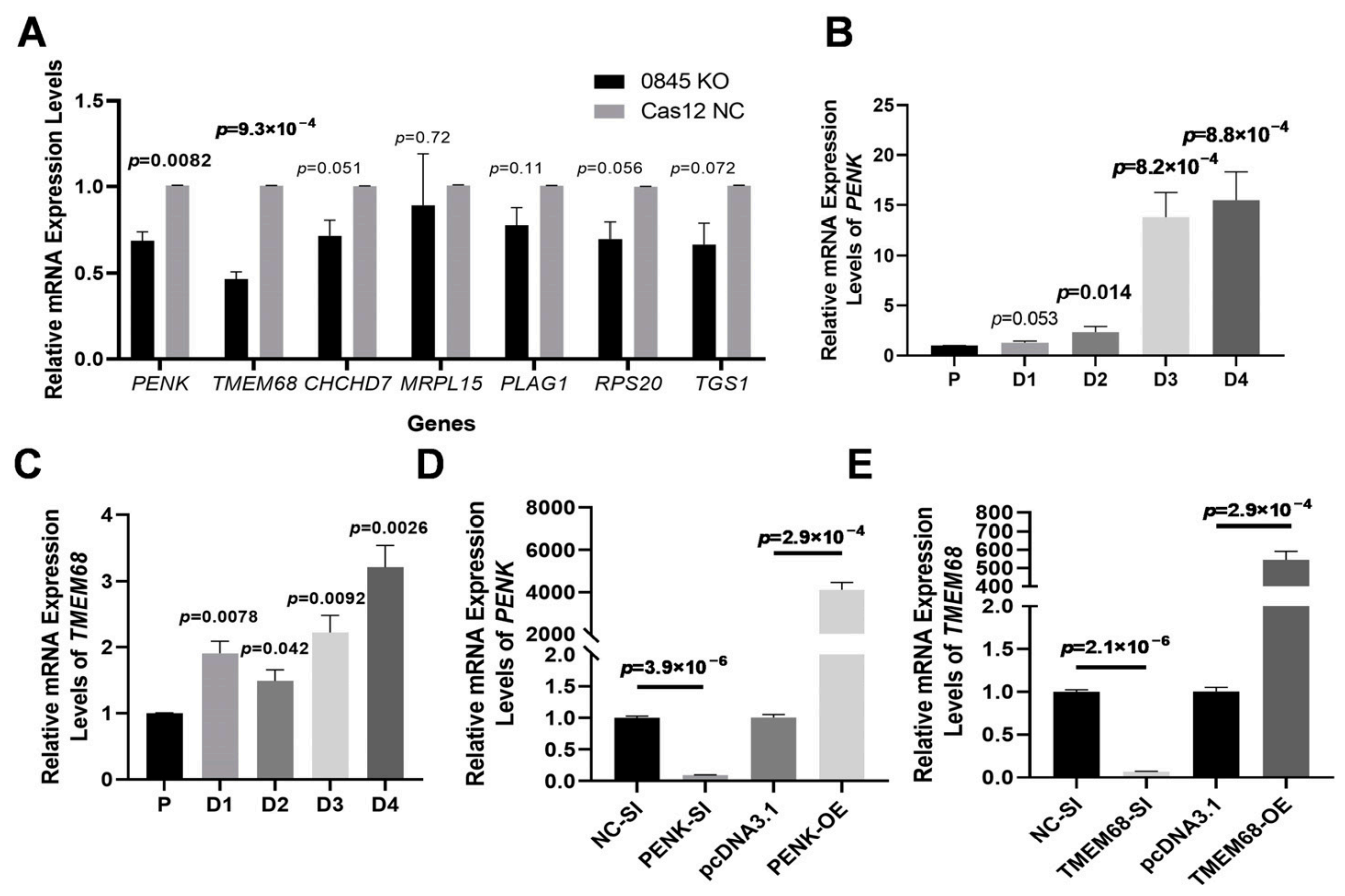
Expression screening identifies *PENK* and *TMEM68* as candidate effector genes downstream of the region containing SNP-0845. (**A**) Quantitative polymerase chain reaction (qPCR) analysis of changes in mRNA levels following deletion of the region containing SNP-0845. Expression levels were normalized to *RN18S1* (*n* = 4). Eight candidate genes were initially examined. *LYN* was not included in the quantitative comparison because of its very low expression in BMSCs (Cq > 35 under the experimental conditions), which precluded reliable relative quantification. (**B**,**C**) Relative expression dynamics of *PENK* (**B**) and *TMEM68* (**C**) during BMSC proliferation and differentiation. Expression levels were normalized to *GAPDH* and *RN18S1* (*n* = 3). The *p* values indicate comparisons with the proliferation phase (P) using two-sided Student’s *t*-tests. (**D**,**E**) Relative mRNA levels of *PENK* (**D**) and *TMEM68* (**E**) after siRNA-mediated knockdown (SI) or plasmid-based overexpression (OE), as determined by qPCR. Expression levels were normalized to *GAPDH* (*n* = 3). P, proliferation phase; D1–D4, days 1–4 of differentiation; PENK-SI, *PENK* knockdown in BMSCs; PENK-OE, *PENK* overexpression in BMSCs; TMEM68-SI, *TMEM68* knockdown in BMSCs; TMEM68-OE, *TMEM68* overexpression in BMSCs.

Our previous muscle expression quantitative trait locus (eQTL) analysis did not identify SNP-0845 as a significant cis-eQTL [4] (Supplementary Table S3). Because cell-type-specific regulatory effects may not be readily detectable in bulk skeletal muscle, we further examined candidate genes prioritized from our previous locus-level eQTL dataset directly in BMSCs. Specifically, we measured their expression by quantitative polymerase chain reaction (qPCR) following deletion of the SNP-0845-containing region. *LYN* showed very low expression in BMSCs, with quantification cycle (Cq) values exceeding 35 under our experimental conditions, and therefore could not be reliably quantified. Among the remaining genes, *PENK* was significantly downregulated in edited cells compared with controls (*p* < 0.01), and *TMEM68* was also significantly decreased (*p* < 0.001; Figure 3A). The other reliably quantified candidate genes did not show significant expression changes following deletion of the SNP-0845-containing region and were therefore not prioritized for subsequent functional and rescue analyses.

To further support the physiological relevance of these candidate genes, we analyzed publicly available bovine transcriptomic data [21]. Both *PENK* and *TMEM68* were detectable in bovine longissimus muscle across newborn, young, and adult stages (Supplementary Figure S4), supporting their expression in the relevant skeletal muscle tissue.

We next examined the expression dynamics of *PENK* and *TMEM68* during BMSC proliferation and myogenic differentiation. Both genes showed increased expression during late differentiation, with significantly higher expression on days 3 and/or 4 of differentiation compared with proliferating cells (Figure 3B,C). These results suggest that *PENK* and *TMEM68* are candidate effector genes associated with the SNP-0845 region and may participate in BMSC fate regulation.

To further investigate the functions of *PENK* and *TMEM68* in BMSCs, small interfering RNA (siRNA)-mediated knockdown and plasmid-mediated overexpression were performed. qPCR analysis confirmed efficient modulation of both genes. *PENK* knockdown reduced *PENK* messenger RNA (mRNA) levels by approximately 90.6%, while *PENK* overexpression increased *PENK* mRNA levels by approximately 4104-fold compared with the corresponding controls. *TMEM68* knockdown reduced its mRNA levels by approximately 93.1%, whereas *TMEM68* overexpression increased *TMEM68* mRNA levels by approximately 544-fold compared with the corresponding controls (Figure 3D,E).

### 2.4. PENK and TMEM68 Regulate BMSC Proliferation, Migration, and Differentiation

**Figure 4:**
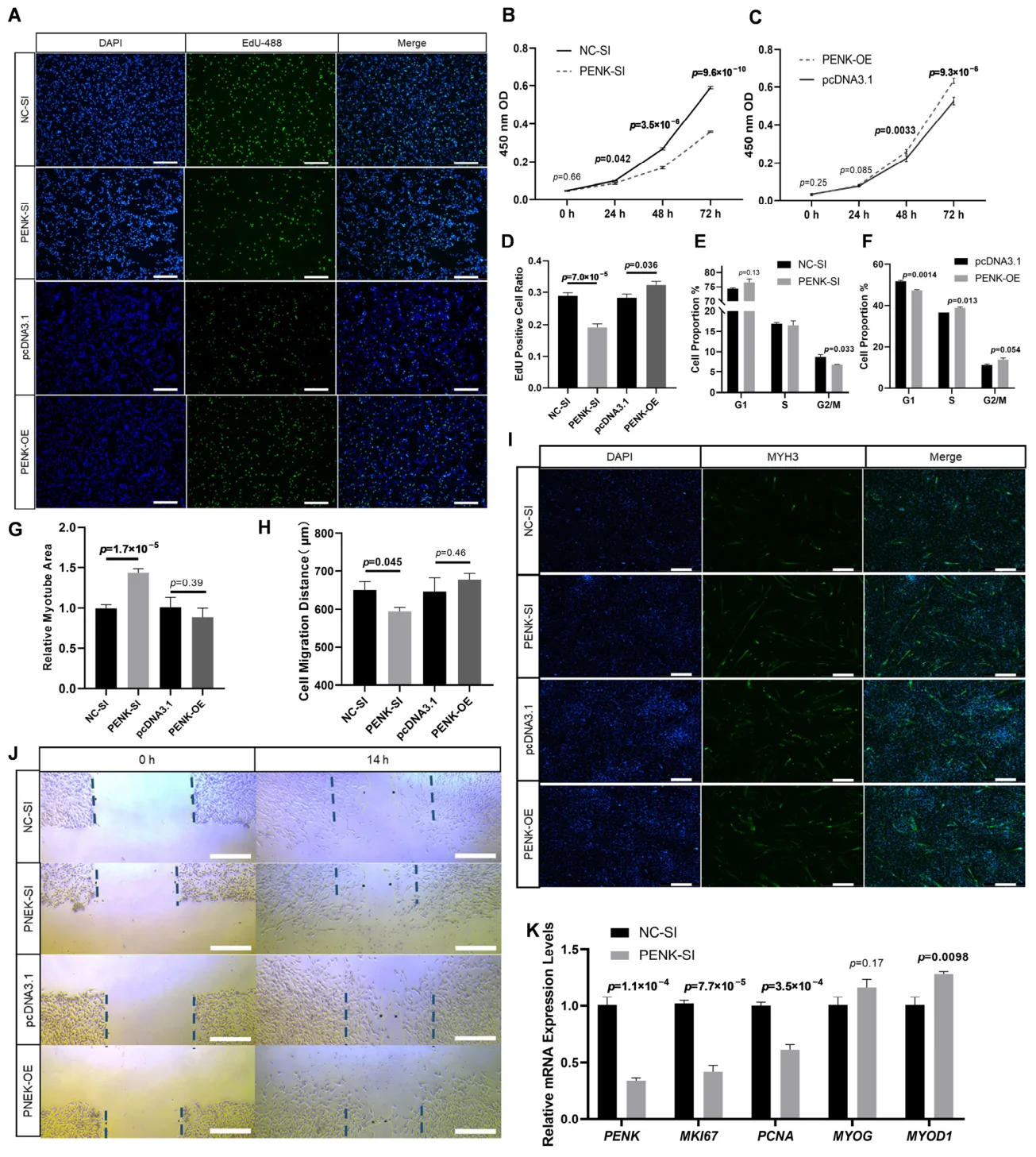
*PENK* promotes BMSC proliferation and migration while inhibiting myogenic differentiation. (**A**) Representative EdU incorporation images showing BMSC proliferation after *PENK* knockdown or overexpression. (**B**,**C**) CCK-8 assays assessing the effects of *PENK* knockdown (**B**) or overexpression (**C**) on BMSC proliferation (*n* = 5). (**D**) Quantification of EdU-positive cells from the experiments shown in (**A**) (*n* = 5). (**E**,**F**) Cell-cycle analysis by flow cytometry following *PENK* knockdown (**E**) or overexpression (**F**) in BMSCs (*n* = 3). (**G**) Quantification of myotube area normalized to nuclei number from the experiments shown in (**I**) (*n* = 6). (**H**) Quantification of wound-healing assays from the experiments shown in (**J**) (*n* = 6). (**I**) MYH3 immunofluorescence staining of myotubes after 4 days of differentiation following *PENK* knockdown or overexpression in BMSCs. (**J**) Representative images of wound-healing assays following *PENK* knockdown or overexpression. Blue dashed lines outline the wound edge at 0 h. (**K**) qPCR analysis of *MKI67*, *PCNA*, *MYOD1*, and *MYOG* mRNA levels following *PENK* knockdown in BMSCs (*n* = 4). Expression levels were normalized to *RN18S1* and *GAPDH*. Scale bars: 400 μm (**A**,**I**,**J**). NC-SI, BMSCs transfected with negative control siRNA; PENK-SI, *PENK* knockdown in BMSCs; pcDNA3.1, empty vector control; PENK-OE, *PENK* overexpression in BMSCs.

After confirming efficient *PENK* knockdown and overexpression (Figure 3D), we further investigated the effects of *PENK* perturbation on BMSC proliferation, cell-cycle progression, myogenic differentiation, and migration. *PENK* knockdown significantly reduced BMSC proliferation, as indicated by reduced EdU incorporation (*p* < 0.001; Figure 4A,D) and decreased CCK-8 absorbance (*p* < 0.001; Figure 4B). Conversely, *PENK* overexpression increased cell viability (*p* < 0.001; Figure 4C) and EdU-positive cell proportion (*p* < 0.05; Figure 4A,D). Cell-cycle analysis further supported the role of *PENK* in BMSC proliferation. *PENK* knockdown altered cell-cycle distribution, whereas *PENK* overexpression decreased the proportion of cells in G1 phase and increased the S-phase population (Figure 4E,F). These results suggest that *PENK* positively regulates BMSC proliferation.

We next examined the effects of *PENK* on myogenic differentiation and migration. *PENK* knockdown significantly enhanced MYH3-positive myotube formation, as reflected by increased myotube area normalized to nuclei number (*p* < 0.05; Figure 4G,I), whereas *PENK* overexpression did not significantly affect differentiation. In wound-healing assays, *PENK* knockdown significantly reduced BMSC migration (*p* < 0.05), whereas *PENK* overexpression showed no significant effect (Figure 4H,J).

Also, *PENK* knockdown significantly reduced the mRNA levels of the proliferation-associated markers *PCNA* and *MKI67* and increased *MYOD1* mRNA levels (Figure 4K). Together, these results suggest that *PENK* supports BMSC proliferation and migration, while its knockdown promotes a shift toward myogenic differentiation. *TMEM68* knockdown significantly reduced BMSC proliferation (EdU-positive cells decreased by 12.99%, *p* < 0.01) and migration (*p* < 0.05), and increased MYH3-positive myotube area by 27.85% (*p* < 0.01). Conversely, *TMEM68* overexpression significantly enhanced migration (*p* < 0.05) but had no significant effect on EdU incorporation or myotube formation, indicating that *TMEM68* contributed to the regulation of BMSC proliferation, migration, and differentiation with generally weaker effects than *PENK* (Supplementary Figure S5).

### 2.5. PENK Shows Stronger Rescue Effects than TMEM68 in BMSCs with Deletion of the SNP-0845-Containing Region

To determine whether the cellular effects caused by deletion of the region containing SNP-0845 were mediated by *PENK* and/or *TMEM68*, we overexpressed *PENK* or *TMEM68* in BMSCs with deletion of the SNP-0845-containing region and examined the resulting changes in proliferation, differentiation, and migration.

**Figure 5:**
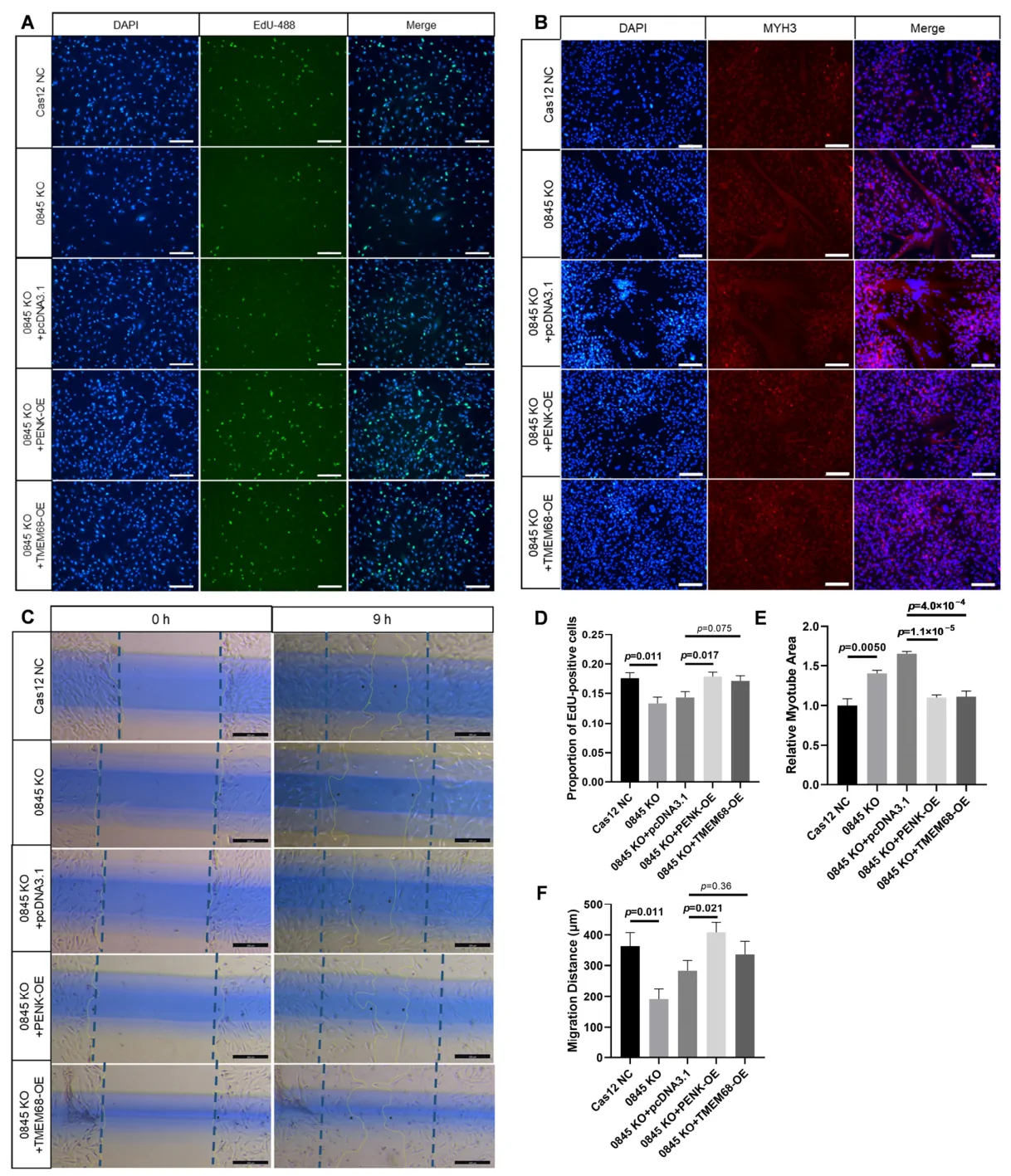
*PENK* and *TMEM68* rescue cellular defects caused by deletion of the region containing SNP-0845 in BMSCs. (**A**) Representative EdU incorporation images of BMSCs with deletion of the region containing SNP-0845 and subsequent overexpression of *PENK* or *TMEM68*. (**B**) Representative MYH3 immunofluorescence images of differentiated BMSCs with deletion of the region containing SNP-0845 and subsequent overexpression of *PENK* or *TMEM68*. (**C**) Representative wound-healing images of BMSCs with deletion of the region containing SNP-0845 and subsequent overexpression of *PENK* or *TMEM68*. (**D**) Quantification of EdU-positive cells from the experiments shown in (**A**) (*n* = 6). (**E**) Quantification of myotube area normalized to nuclei number from the experiments shown in (**B**) (*n* = 4). (**F**) Quantification of migration distance from the wound-healing assays shown in (**C**) after 9 h of migration (*n* = 6). Scale bars: 200 μm (**A**–**C**). Cas12 NC, BMSCs transfected with hfCas12MAX and control crRNA backbone plasmid; 0845 KO, BMSCs with deletion of the region containing SNP-0845; 0845 KO + pcDNA3.1, empty vector control; 0845 KO + PENK-OE, *PENK* overexpression in BMSCs with deletion of the SNP-0845-containing region; 0845 KO + TMEM68-OE, *TMEM68* overexpression in BMSCs with deletion of the SNP-0845-containing region.

EdU assays showed that deletion of the region containing SNP-0845 significantly reduced the proportion of EdU-positive cells compared with the control group (*p* < 0.05), consistent with the impaired proliferative phenotype observed above (Figure 5A,D). In BMSCs with deletion of the SNP-0845-containing region, *PENK* overexpression significantly increased the proportion of EdU-positive cells compared with the vector control (*p* < 0.05), indicating partial rescue of the proliferation defect. In contrast, *TMEM68* overexpression showed a modest increase, but the effect did not reach statistical significance (*p* > 0.05).

We next examined whether *PENK* or *TMEM68* could rescue the altered differentiation phenotype caused by deletion of the region containing SNP-0845. Consistent with previous results, deletion of the region containing SNP-0845 significantly enhanced myotube formation compared with the control group (*p* < 0.01; Figure 5B,E). Overexpression of either *PENK* or *TMEM68* in BMSCs with deletion of the SNP-0845-containing region significantly reduced MYH3-positive myotube formation compared with the vector control (both *p* < 0.001), indicating that both genes could counteract the enhanced differentiation phenotype caused by deletion of the region containing SNP-0845.

Wound-healing assays further showed that deletion of the region containing SNP-0845 significantly reduced cell migration compared with the control group (*p* < 0.05; Figure 5C,F). *PENK* overexpression significantly increased migration distance in BMSCs with deletion of the SNP-0845-containing region (*p* < 0.05), whereas *TMEM68* overexpression resulted in a slight increase that was not statistically significant (*p* > 0.05).

Together, these results indicate that both *PENK* and *TMEM68* contribute to the downstream effects of the region containing SNP-0845, particularly in the regulation of myogenic differentiation. However, *PENK* showed stronger rescue effects on the proliferation and migration defects caused by deletion of the region containing SNP-0845, and was therefore prioritized for subsequent mechanistic investigation.

Because *PENK* encodes a precursor of bioactive peptides [22], we further examined whether *PENK*-derived products exert paracrine effects on neighboring BMSCs. Transwell co-culture and conditioned-medium assays did not support a strong paracrine rescue effect under the current experimental conditions (Supplementary Figure S6), suggesting that *PENK* may regulate BMSC function mainly through a cell-associated or cell-autonomous mechanism.

### 2.6. Penk Knockdown Alters Muscle Growth-Related Phenotypes and Muscle Fiber Composition in Mice

**Figure 6:**
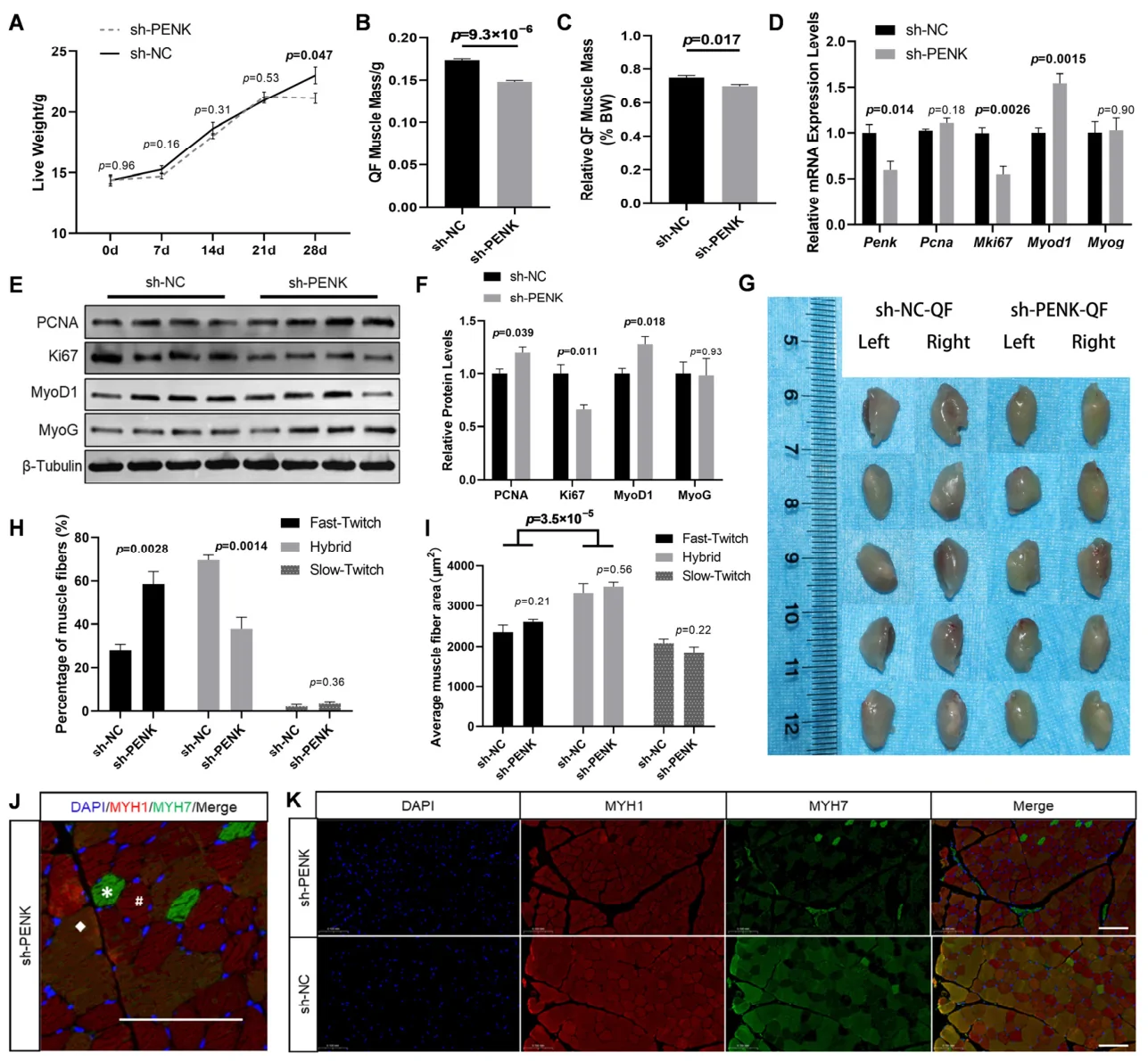
In vivo knockdown of *Penk* reduces quadriceps femoris muscle mass and alters muscle fiber composition in mice. (**A**) Body weight changes in mice during the 28-day experimental period (*n* = 5). (**B**) Absolute quadriceps femoris (QF) muscle weight 28 days after AAV injection (*n* = 5). (**C**) QF muscle weight normalized to body weight 28 days after AAV injection (*n* = 5). (**D**) qPCR analysis of *Penk*, *Pcna*, *Mki67*, *Myod1*, and *Myog* mRNA levels in QF muscle 28 days after intramuscular AAV injection. Expression levels were normalized to *Gapdh* (*n* = 5). (**E**) Western blot analysis of protein levels in QF muscle 28 days after AAV injection. Using β-tubulin as the reference, the reference and target proteins were analyzed on separate gels. However, all samples loaded were derived from the same homogeneous stock. (**F**) Quantification of the Western blot results shown in (**E**). Protein levels were normalized to β-tubulin (*n* = 4). (**G**) Representative photographs of dissected QF muscles from control and sh-PENK mice. The ruler indicates a length range of 5–12 cm. (**H**) Quantification of the proportions of different muscle fiber types (*n* = 4). (**I**) Quantification of the mean cross-sectional area of different muscle fiber types (*n* = 4). (**J**) Schematic classification of different muscle fiber types in this study. Asterisks (*) indicate slow fibers, hashtags (#) indicate fast fibers, and diamonds (◆) indicate hybrid fibers. (**K**) Representative immunofluorescence images of fast fibers (MYH1 staining) and slow fibers (MYH7 staining) after *Penk* knockdown in mouse QF muscle. Scale bars: 100 μm (**J**,**K**). QF, quadriceps femoris muscle; AAV, adeno-associated virus; sh-PENK, AAV-mediated *Penk* knockdown.

To further evaluate the role of *PENK* in muscle growth-related processes in vivo, we generated a *Penk*-knockdown mouse model by intramuscular injection of adeno-associated virus (AAV) expressing short hairpin RNA (shRNA) targeting *Penk* into the quadriceps femoris (QF) muscle. Control mice received sh-NC-AAV. Body weight was recorded weekly for 4 weeks after injection. During the first 3 weeks, no significant difference in body weight was observed between the two groups. At week 4, the mean body weight was 21.17 ± 0.44 g in the sh-PENK group and 23.04 ± 0.66 g in the sh-NC group (mean ± SEM), with a significant reduction in the sh-PENK group (*p* < 0.05; Figure 6A).

At the end of the experiment, mice were euthanized, and QF muscles were collected for phenotypic and molecular analyses. Compared with the control group, QF muscle weight was significantly reduced in the sh-*PENK* group, including both absolute muscle weight (*p* < 0.001; Figure 6B) and body-weight-normalized muscle weight (*p* < 0.05; Figure 6C).

We next examined the mRNA levels of *Penk* and several proliferation- and myogenesis-associated genes in QF muscle. qPCR analysis confirmed that *Penk* mRNA levels was significantly reduced in the sh-PENK group (*p* < 0.05; Figure 6D). *Ki67* mRNA levels was also significantly decreased after *Penk* knockdown (*p* < 0.01), whereas *Myod1* mRNA levels was significantly increased (*p* < 0.01). In contrast, no significant changes were observed in *Pcna* or *Myog* mRNA levels (Figure 6D). At the protein level, Ki67 was similarly decreased and MyoD1 was increased, whereas PCNA protein increased despite the absence of a significant change in *Pcna* mRNA (*p* < 0.05). MyoG remained unchanged (*p* > 0.05; Figure 6E,F). These results suggest that *Penk* knockdown affects the expression of proliferation- and myogenesis-associated markers in QF muscle. Representative photographs of dissected QF muscles are shown in Figure 6G.

To further determine whether *Penk* knockdown influenced muscle fiber composition, QF sections were stained for MYH1 and MYH7 to distinguish fast, slow, and hybrid fibers. Hybrid fibers were defined as fibers positive for both MYH1 and MYH7. Compared with the control group, the sh-*PENK* group showed a significantly higher proportion of fast fibers (*p* < 0.05) and a significantly lower proportion of hybrid fibers (*p* < 0.05), whereas the proportion of slow fibers remained largely unchanged (Figure 6H). We then quantified the mean cross-sectional area of different fiber types. No significant differences in the average area of fast, slow, or hybrid fibers were observed between the sh-*PENK* and control groups (*p* > 0.05). However, across groups, hybrid fibers showed a larger average area than fast fibers (*p* < 0.05; Figure 6I–K).

Taken together, these results indicate that *Penk* knockdown reduces QF muscle weight, alters the expression of muscle growth-related markers, and shifts muscle fiber composition toward a higher proportion of fast fibers without significantly changing the average cross-sectional area of individual fiber types. These in vivo findings support a role for *Penk* in quadriceps growth-related processes and muscle fiber-type homeostasis.

## 3. Discussion

In this study, we functionally characterized two candidate noncoding variants within the *XKR4-CHCHD7* locus that were identified in our previous GWAS-based analysis. Dual-luciferase reporter assays showed that SNP-0845 and SNP-9212 exhibited consistent allele-specific regulatory activity in BMSCs, 293T cells, and C2C12 cells, supporting their potential roles as enhancer-like regulatory variants, consistent with the notion that noncoding variants can influence complex traits by altering cis-regulatory elements and gene expression [23]. Preliminary EMSA further revealed a more distinctive DNA–protein binding pattern at SNP-0845, with an additional shifted band that was not observed for SNP-9212. Although the specificity of this interaction remains to be further validated, this observation provided additional support for prioritizing the SNP-0845-containing region. Subsequent endogenous deletion experiments further strengthened this prioritization, as deletion of the SNP-0845-containing region caused broader and stronger effects on BMSC proliferation, migration, and differentiation than deletion of the SNP-9212-containing region. These findings provided the primary basis for prioritizing the SNP-0845-containing region for subsequent mechanistic investigation.

Deletion of the region containing SNP-0845 reduced the mRNA levels of two nearby candidate effector genes, *PENK* and *TMEM68*, in BMSCs. SNP-0845 is located approximately 701 kb from the *PENK* transcription start site and 193 kb from the *TMEM68* transcription start site, consistent with the possibility that this region participates in distal transcriptional regulation [24]. Although SNP-0845 was not detected as a significant cis-eQTL in our previous bulk-muscle dataset [4], direct perturbation of the SNP-0845-containing region in primary BMSCs significantly reduced *PENK* and *TMEM68* mRNA levels. This difference may reflect the cell-type-dependent nature of regulatory effects that can be diluted in heterogeneous bulk tissues.

Although both *PENK* and *TMEM68* responded to deletion of the SNP-0845-containing region (Figure 3A) and both contributed to its downstream effects on myogenic differentiation (Figure 5B,E), *PENK* showed stronger rescue effects on BMSC proliferation and migration defects than did *TMEM68* (Figure 5A,C,D,F). These results support a phenotype-specific hierarchy rather than an exclusively *PENK*-mediated mechanism: both *PENK* and *TMEM68* may contribute to the differentiation phenotype, whereas *PENK* appears to be the better-supported mediator of the proliferation and migration effects. Thus, the current data support *PENK* as the major candidate effector of the SNP-0845-containing region, while *TMEM68* may act as a secondary, phenotype-dependent component of a broader regulatory mechanism.

Previous studies have implicated *PENK* in muscle related traits, although its function in bovine muscle satellite cells has not been characterized. For example, *PENK* has been associated with longissimus dorsi muscle weight in GWAS-based analyses [19], and differential *PENK* expression has been reported in the longissimus dorsi muscle between lean and obese pig breeds [20]. *PENK* encodes the proenkephalin precursor protein, which can be processed into multiple bioactive enkephalin peptides. Although *PENK* has been extensively studied in neural and endocrine contexts, its role in skeletal muscle development and satellite cell fate regulation remains poorly understood. In the present study, *PENK* was functionally linked to a cattle trait-associated noncoding regulatory region, and direct perturbation demonstrated its involvement in BMSC proliferation, migration, and myogenic differentiation. Together with its detectable expression in bovine skeletal muscle and dynamic expression during BMSC differentiation, these findings extend previous association- and expression-based observations toward functional characterization of *PENK* in bovine muscle satellite cells.

The downstream mechanism by which *PENK* regulates BMSC fate remains unresolved. *PENK*-derived enkephalins can be produced and released by skeletal muscle [17], suggesting a potential extracellular signaling mechanism. In chicken myoblasts, classical opioid receptor transcripts were detected at very low levels, whereas *OGFR* and *OGFRL1* were expressed, and exogenous methionine- and leucine-enkephalin suppressed proliferation [18]. These observations suggest that extracellular *PENK*-derived peptides can influence myogenic-cell behavior, although the relevant receptors and signaling mechanisms in bovine BMSCs remain unknown.

In the present study, both conditioned-medium transfer and Transwell co-culture altered the phenotypes of *PENK*-deficient recipient BMSCs, indicating that the conditioned extracellular environment itself can influence BMSC behavior. However, the effects did not differ significantly according to whether the donor cells expressed low or high levels of *PENK*. One possible explanation is myogenic cells release diverse growth factors, cytokines, and other extracellular proteins that can influence proliferation, migration, and differentiation [25,26,27]; therefore, the responses observed after conditioned-medium transfer or co-culture may instead reflect the combined effects of multiple secreted factors.

The absence of a detectable *PENK*-dependent difference in these assays does not exclude extracellular or cell-associated actions of *PENK*. Changes in *PENK* transcript abundance may not translate proportionally into extracellular concentrations of bioactive peptides because proenkephalin requires post-translational processing to generate mature enkephalins [28]. In addition, proenkephalin has previously been reported to exhibit intracellular/nuclear localization in association with growth-arrest and differentiation responses [29], raising the possibility of functions independent of classical extracellular opioid signaling. Future studies directly measuring *PENK*-derived peptides and examining their processing, receptor dependence, subcellular localization, and downstream signaling will be required to distinguish among these possibilities.

The effect of *PENK* on cell proliferation appears to be context dependent. Previous studies have reported roles for *PENK* or *PENK*-derived peptides in cancer progression [30], developing tissues [15], and myogenic cells [18]. In chicken embryonic myoblasts, exogenous methionine-enkephalin or leucine-enkephalin was reported to suppress proliferation [18], whereas our siRNA-based loss-of-function experiments showed that *PENK* knockdown reduced BMSC proliferation. This difference may reflect species- and cell-type-specific regulation, differences between endogenous *PENK* depletion and exogenous peptide treatment, or concentration-dependent effects of *PENK*-derived peptides. In addition, *PENK* mRNA levels increased during late BMSC differentiation in our study, suggesting that *PENK* may participate in stage-dependent regulation of the proliferation-to-differentiation transition rather than acting as a simple unidirectional promoter or inhibitor of differentiation.

In vivo *Penk* knockdown further supported its role in skeletal muscle growth-related processes. Previous studies reported reduced body weight in *Penk* knockout mice, largely attributing this phenotype to altered feeding behavior [31]. In our study, local AAV-mediated *Penk* knockdown in QF reduced muscle weight and altered muscle growth-related marker mRNA and protein levels, including decreased *Mki67*/Ki67 and increased *Myod1*/MyoD1, while *Myog*/MyoG remained largely unchanged. These findings are generally consistent with the cellular phenotypes observed in *PENK*-deficient BMSCs and suggest that *Penk* may influence skeletal muscle physiology in addition to its known systemic effects. Moreover, *Penk* knockdown altered muscle fiber composition, increasing the proportion of fast fibers and reducing hybrid fibers without significantly changing the average area of individual fiber types. Because skeletal muscle fiber composition is closely related to muscle metabolism [32], contractile properties, and meat quality traits, these results suggest a potential link between *Penk* and muscle fiber-type homeostasis. However, the relevance of these changes to livestock production traits remains to be established.

Some molecular responses were concordant between cultured BMSCs and mouse muscle, including decreased *MKI67*/*Mki67* and increased *MYOD1*/*Myod1* following *PENK*/*Penk* knockdown, whereas others differed between the two systems. In particular, *Pcna* mRNA was not significantly altered in mouse muscle, whereas Pcna protein levels increased. Such discrepancies may reflect species-specific effects, the heterogeneous cellular composition of whole muscle compared with cultured BMSCs [33], differences in differentiation state, or differences between transient siRNA-mediated and sustained AAV-shRNA-mediated knockdown [34,35]. Post-transcriptional regulation may also contribute to the discordance between *Pcna* mRNA and protein levels. It should be noted that the AAV experiment directly manipulated *Penk* rather than SNP-0845 and was conducted using a single AAV dose. Therefore, these results are best interpreted as supportive in vivo evidence for a role of reduced *Penk* expression in skeletal muscle, while future studies using larger cohorts and dose-ranging designs may further establish the robustness of these effects.

Functional validation of noncoding variants remains challenging, particularly in livestock primary cells, where genome-editing efficiency and cell-expansion capacity can be limiting. In this study, CRISPR/hfCas12MAX-mediated deletion allowed us to assess the endogenous function of candidate regulatory regions in primary BMSCs. Compared with reporter assays alone, endogenous perturbation provides complementary and stronger functional evidence by testing regulatory regions within their native genomic context [36]. Similar strategies have been used to validate regulatory variants in other systems, such as genome editing of noncoding regions affecting *FGF13* [37] or *TBX3* [38] expression and related phenotypes. The consistent direction of the phenotypic effects across multiple complementary assays, including proliferation, cell-cycle progression, migration, and myogenic differentiation, supports the biological relevance of the SNP-0845-containing region. Such quantitative effects are consistent with the regulatory nature of cis-regulatory elements, whose endogenous perturbation can produce biologically meaningful changes in gene expression and cellular phenotypes [39,40].

Our CRISPR strategy established the functional relevance of the genomic region containing SNP-0845 in its endogenous context. Because the present approach involved regional deletion rather than precise nucleotide substitution, future allele-specific editing will help resolve the contribution of SNP-0845 at single-nucleotide resolution. The preliminary EMSA further supported differential nuclear-protein binding at the SNP-0845-containing sequence, and future competition and supershift experiments will help define the sequence specificity of this interaction and identify the relevant DNA-binding factor(s). Together, these approaches will further refine the regulatory mechanism at this locus. Subject to such validation, SNP-0845 may represent a candidate functional variant of potential interest for cattle genomic improvement. Previous studies have shown that prioritization of functionally supported sequence variants can contribute to genomic prediction and the design of informative genotyping panels [41], although the predictive value and stability of SNP-0845 across cattle populations remain to be determined. Overall, our findings establish the SNP-0845-containing region as a functionally relevant regulatory region in bovine muscle satellite cells and identify *PENK* as its major candidate effector, while also supporting a secondary, phenotype-dependent contribution of *TMEM68*.

## 4. Materials and Methods

### 4.1. Cell Culture

Primary bovine muscle satellite cells (BMSCs) were independently isolated from approximately 10 bovine fetuses at approximately 3 months of gestation, obtained from pregnant cattle at a local slaughterhouse. Approximately 1 g of longissimus dorsi muscle was collected from each fetus. After removal of visible connective tissue, the muscle samples were washed, minced into small pieces, and digested with type I collagenase as previously described [42]. The isolated cells were collected and cultured in Dulbecco’s modified Eagle medium (DMEM, Gibco, Grand Island, NY, USA) supplemented with 20% fetal bovine serum (FBS, Gibco, Grand Island, NY, USA) and 1% penicillin–streptomycin (Gibco, Grand Island, NY, USA). Cells derived from different fetuses were maintained separately and used as independent primary BMSC preparations in subsequent experiments.

HEK-293T (CRL-3216) and C2C12 (CRL-1772) cells were obtained from the American Type Culture Collection (ATCC, Manassas, VA, USA) and cultured in DMEM supplemented with 10% FBS and 1% penicillin–streptomycin. Myogenic differentiation of BMSCs and C2C12 cells was induced at approximately 70% confluence by replacing the growth medium with DMEM supplemented with 4% horse serum and 1% penicillin–streptomycin. Culture dishes used for BMSCs were precoated with laminin-521 (Thermo Fisher Scientific, Waltham, MA, USA). All cells were maintained at 37 °C in a humidified atmosphere containing 5% CO_2_.

### 4.2. Plasmid Construction and Small Interfering RNA Synthesis

For construction of the dual-luciferase reporter plasmids, genomic fragments of approximately 500 bp centered on each candidate SNP were amplified from bovine genomic DNA using 2× Phanta Max Master Mix (Dye Plus) (Vazyme, Nanjing, China) and the corresponding primers listed in Supplementary Table S4. The annealing temperature was 64 °C. Polymerase chain reaction (PCR) products were purified using the E.Z.N.A. Gel Extraction Kit (Omega, Norcross, GA, USA) and inserted into the KpnI- and MluI-digested pGL4.23 vector using 2× MultiF Seamless Assembly Mix (ABclonal, Wuhan, China). The alternative alleles at each SNP were subsequently introduced by site-directed mutagenesis using the Mut Express MultiS Fast Mutagenesis Kit V2 (Vazyme, Nanjing, China) and the corresponding mutagenic primers (Supplementary Table S4).

For construction of the *PENK* and *TMEM68* overexpression plasmids (PENK-OE and TMEM68-OE), the coding sequence (CDS) of bovine *PENK* and *TMEM68* were amplified from bovine RNA-reverse transcribed complementary DNA (cDNA) using 2×Phanta Max Master Mix (Dye Plus) and corresponding CDS amplification primers (Supplementary Table S4). The pcDNA3.1(+) overexpression vector was linearized by PCR using the pc3.1-MYC-F/R primers (Supplementary Table S4). The amplified CDS fragments were inserted into the linearized pcDNA3.1(+) vector using 2× MultiF Seamless Assembly Mix, followed by transformation into competent cells.

For construction of the CRISPR RNA (crRNA) plasmid used in the CRISPR/hfCas12MAX editing system, the hfCas12MAX [43] was first digested with BbsI (Takara, Kusatsu, Shiga, Japan) for 3 h, and the linearized plasmid was purified using the E.Z.N.A. Gel Extraction Kit (Omega, Norcross, GA, USA). Forward and reverse crRNA oligonucleotides (Supplementary Table S4) were mixed with 10× PCR buffer at a ratio of 9:9:2, annealed, and subsequently diluted 200-fold. The annealed oligonucleotides were ligated into the linearized hfCas12MAX plasmid using the DNA Ligation Kit (Takara, Kusatsu, Shiga, Japan), followed by transformation into DH5α competent cells (Vazyme, Nanjing, China). Individual colonies were selected for plasmid preparation and verification.

All plasmids were purified from 16 h bacterial cultures using the E.Z.N.A. Endo-Free Plasmid DNA Mini Kit II (Omega, Norcross, GA, USA) and verified by Sangon Biotech (Beijing, China) through Sanger sequencing.

The siRNAs of *PENK* and *TMEM68* were designed and synthesized by GenePharma (Shanghai, China). The siRNA sequences are provided in Supplementary Table S5. Lyophilized siRNAs were resuspended in 125 µL of RNase-free ddH_2_O to obtain a final stock concentration of 20 µM, aliquoted, and stored at −20 °C to avoid repeated freeze–thaw cycles.

### 4.3. Dual-Luciferase Reporter Assay

Cells were seeded in 24-well plates and cultured until they reached approximately 80% confluence. Each well was then co-transfected with 500 ng of the recombinant vector (pGL4.23, SNP-0845-A, SNP-0845-G, SNP-9212-T, SNP-9212-G, etc.; Supplementary Table S4), and 50 ng of pGL4.74 hRluc/Tk internal control plasmid using Lipofectamine 3000 (Thermo Fisher Scientific, Waltham, MA, USA). After 48 h, cells were lysed, and firefly and *Renilla* luciferase activities were measured using the Dual-Glo Luciferase Assay System (Promega, Madison, WI, USA) according to the manufacturer’s instructions. The Luminescence was measured using an Infinite 200 PRO (TECAN, Männedorf, Switzerland). Relative transcriptional activity was calculated as the ratio of firefly luciferase activity to *Renilla* luciferase activity. Each condition was assessed in three technical replicate wells, and the assay was independently repeated in four experimental runs on separate days using the same cell preparation.

### 4.4. Electrophoretic Mobility Shift Assay

Nuclear proteins were extracted from approximately 5 × 10^6^ BMSCs per sample using a nuclear and cytoplasmic protein extraction kit (Beyotime, Shanghai, China). Nuclear extracts obtained from three independent BMSC preparations (BMSCs-1, BMSCs-2, and BMSCs-3) were pooled in equal amounts for the subsequent electrophoretic mobility shift assay (EMSA). Biotin-labeled forward and reverse oligonucleotides (10 µM; Supplementary Table S4) were annealed in a total volume of 120 µL containing 18 µL of each oligonucleotide and 84 µL of annealing buffer. The mixture was heated at 97 °C for 2 min and then allowed to cool to room temperature for annealing. Binding reactions containing 5 nM biotin-labeled probe and nuclear extract at a final protein concentration of 0.6 µg/µL were incubated at room temperature for 20 min. The reaction mixtures were then combined with loading buffer and separated on a 6% native polyacrylamide gel. Before sample loading, the gel was pre-run at 100 V for 10–40 min. Electrophoresis was performed at 100 V until the dye front reached approximately two-thirds of the gel. The gel was transferred to a nylon membrane pre-wetted in 0.5×Tris-borate-EDTA (TBE) at 100 V for 1 h, followed by ultraviolet (UV) crosslinking for 30 s. The membrane was blocked for 30 min, incubated with horseradish peroxidase (HRP) -conjugated streptavidin (1:2000) for 30 min, and washed. Chemiluminescent signals were detected after addition of enhanced chemiluminescence (ECL) reagent (Beyotime, Shanghai, China).

A free-probe control containing the kit positive-control probe without protein and a positive binding control containing the same probe together with the kit-provided positive-control protein were included to verify probe migration and assay performance, respectively.

### 4.5. In Silico Transcription Factor-Binding Motif Analysis

Potential transcription factor-binding motifs affected by SNP-0845 and SNP-9212 were analyzed using the JASPAR 2026 database (https://jaspar.elixir.no/) [44]. For each variant, two 21 bp sequences containing 10 bp of flanking sequence on either side of the SNP were analyzed using the JASPAR CORE collection with *Homo sapiens* transcription factor profiles and a relative profile score threshold of 80%. Only predicted motifs overlapping the variant position were considered. For each SNP, the five motifs with the highest relative scores were selected for further allele-specific comparison. The relative score of the corresponding alternative allele was subsequently calculated using the same JASPAR matrix, and the difference in relative score (ΔRelative score) was calculated as the higher relative score minus the alternative-allele score. Representative motifs were visualized using sequence logos reconstructed from the corresponding JASPAR position frequency matrices.

### 4.6. CRISPR/hfCas12MAX-Mediated Deletion of Candidate Regulatory Regions

Approximately 5 × 10^5^ BMSCs were electroporated with 2 μg of the corresponding hfCas12MAX plasmid using a Lonza 4D-Nucleofector system (Lonza, Basel, Switzerland) with program CZ167, according to the manufacturer’s instructions for the P3 Primary Cell Nucleofector Kit (Lonza, Basel, Switzerland). At 48 h after electroporation, green fluorescent protein (GFP)-positive cells were isolated by flow cytometric sorting and subsequently expanded for downstream analyses. Independent primary BMSC preparations were used for separate editing experiments. Most functional assays were performed using bulk populations of GFP-positive edited cells. Monoclonal cell populations were used only for one CCK-8 experiment and for characterization of editing events. For monoclonal cell isolation, individual GFP-positive cells were sorted into 96-well plates by flow cytometry. After approximately 7 days of culture, proliferating clones were transferred to 48-well plates. Cells were subsequently expanded sequentially into 24-well and 12-well plates. Approximately 500 cells from each expanded clone were collected for genotyping, and the remaining cells were cryopreserved when they reached approximately 80% confluence.

To assess editing efficiency in bulk GFP-positive cell populations, approximately 1000 sorted cells were collected and lysed using tissue lysis buffer (Vazyme, Nanjing, China). The target genomic regions were amplified by PCR using the corresponding editing-detection primers. The purified PCR products were cloned into the pUC19 vector using the Ultra-Universal TOPO Cloning Kit (Vazyme, Nanjing, China), followed by transformation into competent cells. Individual bacterial colonies were selected, and the inserted fragments were analyzed by Sanger sequencing (Sangon Biotech, Beijing, China). Editing efficiency was determined based on the sequencing results.

### 4.7. AAV-Mediated Penk Knockdown and Mouse Experiment

For in vivo knockdown targeting mouse *Penk*, an adeno-associated virus serotype 9 (AAV9) vector expressing a short hairpin RNA (shRNA) against *Penk* (sh-PENK-AAV) was designed and synthesized by GenePharma (Shanghai, China). The target sequence was 5′-CCTCCGATCTGCTGAAAGA-3′. The shDNA template oligonucleotides were: sense strand, 5′-ACCGCCTCCGATCTGCTGAAAGATTCAAGAGATCTTTCAGCAGATCGGAGG-3′; antisense strand, 5′-AAACCTCCGATCTGCTGAAAGATCTCTTGAATCTTTCAGCAGATCGGAGGC-3′. The viral titer, determined by quantitative PCR (qPCR), was 2.42 × 10^13^ vector genomes (V.G.)/mL. The negative control AAV (sh-NC-AAV) was designed with a non-targeting sequence (5′-ACTACCGTTGTTATAGGTG-3′) and had a titer of 1.51 × 10^13^ V.G./mL.

Ten healthy 4-week-old male C57BL/6J mice were purchased from Beijing Hua Yuan Shi Dai Technology Co., Ltd. (Beijing, China). After 1 week of acclimatization, the mice were randomly assigned to the sh-PENK-AAV and sh-NC-AAV groups (*n* = 5 per group). AAV preparations were adjusted to a working concentration of 1 × 10^12^ vg/mL, and approximately 50 μL was injected into each quadriceps femoris (QF) muscle at 3–4 intramuscular sites. Following injection, mice had ad libitum access to food and water and were maintained for 28 days, with body weight recorded every 7 days. On day 28, mice were weighed and then euthanized. The bilateral QF muscle was dissected, weighed, and photographed. Muscle length and cross-sectional area were measured from the images using ImageJ software (Version 1.54i). The right QF muscle from each mouse was used for RNA extraction and subsequent qPCR analysis.

### 4.8. RNA Extraction and Quantitative PCR

Total RNA was extracted using TRIzol (Thermo Fisher Scientific, Waltham, MA, USA) according to the manufacturer’s instructions. After measurement of RNA concentration, first-strand cDNA was synthesized using the HiScript II 1st Strand cDNA Synthesis Kit (+gDNA wiper) (Vazyme, Nanjing, China) on a ProFlex^TM^ Base (Applied Biosystems, Calverton, CA, USA). Genomic DNA was first removed at 42 °C for 2 min. Reverse transcription was then performed in a 20-μL reaction at 50 °C for 15 min, followed by enzyme inactivation at 85 °C for 2 min, according to the manufacturer’s instructions. The resulting cDNA was diluted fivefold and used as the template for quantitative PCR (qPCR) with Taq Pro Universal SYBR qPCR Master Mix (Vazyme, Nanjing, China). Reactions were performed on a TSQ-4096T quantitative PCR system (TransGen Biotech, Beijing, China). The cycling conditions were as follows: initial denaturation at 95 °C for 30 s, followed by 40 cycles of denaturation at 95 °C for 10 s and annealing/extension at 60 °C for 30 s. A melting-curve analysis was subsequently performed. Each sample was analyzed in three technical replicate reactions. Primer sequences used for qPCR are listed in Supplementary Table S4. Relative mRNA levels were calculated using the 2^−ΔΔCq^ method [45], with *RN18S1* and/or *GAPDH* used as reference genes as specified for the corresponding experiments. Genes showing consistently late amplification (Cq > 35) under the experimental conditions were considered insufficiently abundant for reliable relative quantification and were excluded from comparative expression analyses.

### 4.9. siRNA or Overexpression Plasmid Transfection

For siRNA-mediated knockdown, BMSCs were transfected with PENK-SI, TMEM68-SI, or NC-SI using Lipofectamine RNAiMAX (Thermo Fisher Scientific, Waltham, MA, USA) at a final concentration of 100 nM. For plasmid-mediated overexpression, BMSCs cultured in 6-well plates were transfected with 2 μg of the PENK-OE or TMEM68-OE plasmid, or the corresponding empty-vector control, using Lipofectamine 3000 according to the manufacturer’s instructions. Knockdown and overexpression efficiencies were evaluated by qPCR 48 h after transfection.

### 4.10. Rescue Assay

To determine whether *PENK* or *TMEM68* could rescue the cellular phenotypes induced by deletion of the SNP-0845-containing region, a sequential genome-editing and overexpression strategy was used. Primary BMSCs were seeded in laminin-521-coated 10 cm culture dishes and cultured until they reached approximately 70–80% confluence. CRISPR/hfCas12MAX-mediated deletion of the region containing SNP-0845 was performed using the Lonza 4D-Nucleofector system with program CZ167, as described above.

One day after electroporation, GFP-positive cells were sorted by flow cytometry and reseeded into 6-well plates. After approximately 24 h of recovery, when cells reached about 70% confluence, transfection was performed as follows. For each well of a 6-well plate, 2 μg of PENK-OE, TMEM68-OE, or empty pcDNA3.1 plasmid was transfected using Lipofectamine 3000 according to the manufacturer’s instructions. After 6 h, 2 mL growth medium was added. Twenty-four hours later, cells were passaged into appropriate plates for EdU incorporation, MYH3 immunofluorescence, and wound-healing assays.

### 4.11. Proliferation Assay (CCK-8 and EdU Assays)

BMSC proliferation was evaluated using Cell Counting Kit-8 (CCK-8) (Vazyme, Nanjing, China) and 5-ethynyl-2′-deoxyuridine (EdU) incorporation assays.

For the CCK-8 assay, cells were seeded in 96-well plates at a density of 2 × 10^3^ cells/well (three technical replicates per biological replicate) at 24 h after transfection. Following incubation for 0 h, 24 h, 48 h and 72 h, cell viability was determined by CCK-8 assays performed according to the manufacturer’s instructions. Absorbance was determined at a wavelength of 450 nm with an iMark Microplate Absorbance Reader (Bio-Rad, Hercules, CA, USA).

To assess the proportion of cells undergoing DNA synthesis, a 5-ethynyl-2′-deoxyuridine (EdU) incorporation assay was performed. At 24 h post-transfection, according to the manufacturer’s protocol of the Cell-Light EdU Apollo488 In Vitro Kit (RiboBio, Guangzhou, China), the cells were labeled with EdU for 2 h using this kit, followed by immediate fluorescence staining. Then we captured 10 random fields (as technical replicates) for each sample using a fluorescence microscope (TCS SP8, Leica, Wetzlar, Germany) and the number of EdU-positive cells and total nuclei was quantified using StarDist (v0.3.0) [46] in ImageJ. The EdU-positive rate was calculated as the number of EdU-positive cells divided by the total number of nuclei.

### 4.12. Cell Cycle Detection

For cell-cycle analysis, BMSCs subjected to siRNA-mediated knockdown or plasmid-mediated overexpression were collected 24 h after transfection. For CRISPR/hfCas12MAX-mediated editing experiments, GFP-positive cells isolated by flow cytometric sorting were cultured for an additional 24 h before analysis. The adherent cells were collected by trypsin digestion and fixed overnight in 70% ethanol at −20 °C freezer with 70% alcohol. After 24 h, they were stained with propidium iodide dye containing RNAse for 30 min at 37 °C. Fluorescence collection was immediately performed using a flow cytometer (FACSVerse, Becton Dickinson, Franklin Lakes, NJ, USA), and cell cycle analysis was performed using ModFit LT (V3.1, Verity Software House, Topsham, ME, USA). Cell-cycle experiments were performed in three independent experimental runs using separately frozen aliquots derived from the same BMSC preparation. One transfection and analysis experiment was performed per day over three separate days.

### 4.13. Migration Assay

For the scratch wound assay, cells were plated into a 12-well plate and then transfected. At 24 h after treatment, a linear scratch was generated in the cell monolayer using a sterile pipette tip, and the culture medium was replaced with medium containing 1% FBS to minimize the contribution of cell proliferation to wound closure. Images of the same wound regions were acquired at 0 and 9 or 14 h using a TCS SP8 microscope (Leica). For each image, wound width was measured at four evenly distributed positions using ImageJ, and the mean wound width was calculated. Migration distance was calculated as D0 − D1, where D0 represents the mean wound width at 0 h and D1 represents the mean wound width at 9 or14 h.

### 4.14. Immunofluorescence

For analysis of BMSC myogenic differentiation, immunofluorescence staining was performed as previously described [47]. Briefly, cells were fixed, permeabilized, blocked, and incubated with a MYH3 polyclonal antibody (1:200, Proteintech, Wuhan, China) at 4 °C for 12 h. After washing with phosphate-buffered saline (PBS) (with 1% bovine serum albumin (BSA)), cells were incubated with AF488-labeled goat anti-rabbit IgG (H + L) (1:200, Beyotime, Shanghai, China) or Cy3-conjugated goat anti-rabbit IgG secondary antibody (1:200, Servicebio, Wuhan, China) at 37 °C for 1 h in the dark. Nuclei were subsequently counterstained with 4′,6-diamidino-2-phenylindole (DAPI) (Thermo Fisher Scientific, Waltham, MA, USA). For each sample, 10 random fields were captured as technical replicates using a fluorescence microscope (TCS SP8, Leica). The MYH3-positive area was quantified using ImageJ, and the number of nuclei in the corresponding images was counted using StarDist [46]. Myogenic differentiation was quantified as the MYH3-positive area normalized to the number of nuclei.

For mouse muscle fiber-type analysis, quadriceps femoris (QF) muscles were fixed in 4% paraformaldehyde, embedded in paraffin, and sectioned. After deparaffinization and rehydration, sections were subjected to antigen retrieval in Tris-EDTA buffer (pH 8.0) at 90 °C for 30 min, followed by natural cooling and washing with PBS three times for 5 min each. Sections were then blocked with 3% BSA at room temperature for 30 min and incubated overnight at 4 °C with a rabbit anti-MYH1 antibody (1:500, Servicebio, Wuhan, China) and a mouse anti-MYH7 antibody (1:500, Servicebio, Wuhan, China). After washing with PBS, sections were incubated with Cy3-conjugated goat anti-rabbit IgG (1:300) and Alexa Fluor 488-conjugated goat anti-mouse IgG (1:400, Servicebio, Wuhan, China) for 50 min at room temperature in the dark. Nuclei were counterstained with DAPI, and sections were mounted with antifade mounting medium.

Whole muscle sections were imaged at the same magnification for all samples. Based on MYH1 and MYH7 immunoreactivity, muscle fibers were classified as fast fibers (MYH1+/MYH7−), slow fibers (MYH1−/MYH7+), or hybrid fibers (MYH1+/MYH7+). ImageJ software was used for threshold-based fiber classification and quantification. Fiber counts from the complete section of each mouse were pooled to calculate a single proportion for each fiber type per animal. Individual mice were then used as the units for statistical analysis.

### 4.15. Western Blotting

Quadriceps femoris (QF) muscle tissues were lysed in ice-cold radioimmunoprecipitation assay (RIPA, Beyotime, Shanghai, China) buffer supplemented with 1 mM phenylmethylsulfonyl fluoride (PMSF, Beyotime, Shanghai, China). Protein concentrations were determined using a bicinchoninic acid (BCA, Beyotime, Shanghai, China) assay. Equal amounts of protein from each sample were mixed with loading buffer and heated at 95–100 °C for 5 min.

Proteins were separated on 4–12% SurePAGE gels and transferred onto nitrocellulose membranes. The membranes were incubated with the corresponding primary antibodies overnight at 4 °C. The primary antibodies and dilutions used were as follows: Ki67 (1:1000, Proteintech, Wuhan, China), MyoD1 (1:2000, Proteintech, Wuhan, China), MyoG (1:500, ABclonal, Wuhan, China), PCNA (1:2000, Proteintech, Wuhan, China), and β-tubulin (1:2000, Proteintech, Wuhan, China). After washing, the membranes were incubated with the appropriate horseradish peroxidase (HRP)-conjugated secondary antibodies (1:2000, Beyotime, Shanghai, China) for 1 h at room temperature. Protein bands were visualized using enhanced chemiluminescence (ECL) reagent and imaged using a Tanon 5200 chemiluminescence imaging system (Shanghai, China). Band intensities were quantified using ImageJ and normalized to β-tubulin.

### 4.16. Paracrine Assay Using Conditioned Medium and Transwell Co-Culture

To evaluate whether *PENK*-derived products regulate BMSC function through paracrine signaling, conditioned-medium and Transwell co-culture assays were performed. For conditioned-medium preparation, PENK-SI or PENK-OE BMSCs were cultured to 70–80% confluence, and supernatants were collected after 24 h. The supernatants were centrifuged at 1000× *g* for 5 min to remove cell debris and used on the same day.

For the Transwell co-culture assay, 12-well Transwell inserts with 0.4-μm pores were used. Transfected BMSCs (1 × 10^4^ cells) were seeded in the lower chamber in 1000 μL of growth medium containing 20% serum, and 5 × 10^3^ cells were seeded in the upper chamber in 200 μL of the same medium. PENK-SI cells in the lower chamber served as recipient cells, whereas PENK-SI or PENK-OE cells in the upper chamber served as donor cells. Four experimental groups were included: NC-SI, PENK-SI, PENK-SI + PENK-SI, and PENK-SI + PENK-OE.

Following conditioned-medium treatment or Transwell co-culture, CCK-8, EdU incorporation, MYH3 immunofluorescence, and wound-healing assays were performed as described above.

### 4.17. Sources of Materials and Consumables

Detailed information on the major reagents, antibodies, cell lines, and corresponding catalog numbers used in this study is provided in Supplementary Table S6.

### 4.18. Statistical Analysis

Publicly available bovine transcriptomic data were obtained from the Cattle BodyMap database reported by Cai et al. [21]. Expression levels of *PENK* and *TMEM68* in longissimus muscle from newborn, young, and adult cattle were extracted and visualized based on reported fragments per kilobase of transcript per million mapped reads (FPKM) values.

Statistical significance was determined by the following: (i) one-way analysis of variance with Tukey’s multiple comparison test was used to compare differences in mean values at the 5% significance level and (ii) a two-sided Student’s *t*-test was utilized for the difference analysis of two contrasts. Unless otherwise indicated, n represents the number of independent biological replicates. Technical replicates within each experiment were averaged and were not treated as independent observations. Data were analyzed and visualized using GraphPad Prism 8 (version 8.0.2, GraphPad Software, Boston, MA, USA). Results are presented as the mean ± standard error of the mean (SEM). A *p* value < 0.05 was considered statistically significant. Exact *p* values are provided in the figures where applicable. Unless otherwise specified, the *p* values shown in the figures represent comparisons between the indicated experimental groups and their corresponding control groups.

## 5. Conclusions

This study comprehensively characterized the function of a candidate noncoding regulatory region within the *XKR4-CHCHD7* locus. This region exhibited allele-specific enhancer-like activity and, within its endogenous genomic context, regulated bovine muscle satellite cell (BMSC) proliferation, migration, and myogenic differentiation. *PENK* and *TMEM68* were identified as candidate effector genes downstream of this region, and rescue experiments further identified *PENK* as a key mediator of its cellular effects. Functional analyses indicated that *PENK* contributes to the regulation of BMSC fate, while in vivo knockdown of *PENK* provided in vivo support for its involvement in muscle growth-related processes and fiber-type homeostasis. Collectively, these findings provide functional evidence that a noncoding regulatory region on BTA14 influences bovine muscle satellite cell function and muscle growth-related phenotypes, at least in part through *PENK*.

## Data Availability

The original contributions presented in this study are included in the article/Supplementary Materials. Further inquiries can be directed to the corresponding authors.

## Supplementary Materials

The following supporting information can be downloaded at: https://www.mdpi.com/article/10.3390/ijms27167467/s1.
